# A Lightweight Key Agreement Protocol Based on Chinese Remainder Theorem and ECDH for Smart Homes

**DOI:** 10.3390/s20051357

**Published:** 2020-03-02

**Authors:** Yi Jiang, Yong Shen, Qingyi Zhu

**Affiliations:** 1 School of Computer Science and Technology, Chongqing University of Posts and Telecommunications, Chongqing 400065, China; jiangyi@cqupt.edu.cn (Y.J.); S170201064@stu.cqupt.edu.cn (Y.S.); 2 School of Cyber Security and Information Law, Chongqing University of Posts and Telecommunications, Chongqing 400065, China

**Keywords:** smart homes, key agreement protocol, Chinese remainder theory, ECDH

## Abstract

Security and efficiency are the two main challenges for designing a smart home system. In this paper, by incorporating Chinese remainder theorem (CRT) into the elliptic curve Diffie–Hellman (ECDH), a lightweight key agreement protocol for smart home systems is constructed. Firstly, one-way hash authentication is used to identify the sensor nodes instead of mutual authentication to reduce the authentication cost. Secondly, the CRT is introduced to enhance the security of the original ECDH key agreement. Security analysis showed that the proposed protocol can validate the data integrity and resist the replay attack, the man-in-middle attack, and other attacks. Performance analysis and experiments showed that the protocol achieves high security with low communication and computation costs, and can be implemented in smart home systems.

## 1. Introduction

The Internet of Things (IoT), ranging from wearable devices [1], smart homes [2], healthcare [3], smart cities [4], and smart agriculture [5], to industrial automation [6], has rapidly developed. With the evolution of IoT technologies, smart home products have been becoming increasingly intelligent and easy to use. For example, via electronic sensors, connected homes are capable of monitoring environmental conditions including lighting, temperature, and motion.

A general IoT architecture model consists of three layers: a sensing layer, a network layer, and an application layer. In the sensing layer, since many IoT devices work in an unattended fashion with no or limited tamper resistance policies and methodologies, an attacker might take advantage of physical access to some devices, leading to significant damage [7]. In the network layer, the wireless communication between sensors might be intercepted and eavesdropped by malicious attackers. The application layer provides communication interfaces for users to control the IoT devices, but a malicious attacker might use devices to act as the legal devices to join to the IoT application network without authentication. Attackers might also collect and analyze the IoT application network traffic, which could pose a threat to the privacy of its users. Similarly, most sensors in smart homes have constrained conditions, such as limited computation ability, small memory storage, low transmission bandwidth, and small battery capacity. Hence, many security issues exist in the smart home environment, including deficient physical security [8], insufficient energy harvesting [9], inadequate authentication [10], and improper encryption [11]. Therefore, a well-designed, secure, and lightweight protocol for smart homes is an imperative.

To secure the communication between IoT devices and servers, many elliptic curve Diffie–Hellman (ECDH)-based key agreement protocols have been proposed [12,13,14,15]. Inspired by previous work, we aimed to improve the security of ECDH by incorporating Chinese remainder theorem (CRT), which is a lightweight parameters negotiation algorithm. Considering the trade-off between security and performance, instead of mutual authentication, one-way authentication is used to prevent the unauthorized devices from accessing the smart home network while meeting the low energy and computation consumption requirements of resource-constrained devices. The novelty and main contributions of this paper are summarized as follows:

(1) To improve the security of ECDH key agreement algorithm, a lightweight CRT is introduced to achieve the parameters negotiation of ECDH. The proposed protocol not only establishes a secure shared key, but also improves operational efficiency and reduces energy consumption.

(2) One-way authentication is proposed to identify the devices, which can significantly reduce the authentication computation cost and prohibit illegal devices from joining the network.

(3) The detailed security analysis showed that our proposed protocol can establish a secure shared key. The performance evaluations proved that our scheme is a lightweight scheme with low computation cost and small memory storage space.

The remainder of this paper is organized as follows: In Section 2, some related work of IoT security protocols is introduced. In Section 3, a brief architecture model for smart home is outlined. Section 4 describes the protocol in detail. The security analysis and performance evaluation of proposed protocol is described in Section 5. Finally, the conclusions are provided in Section 6.

## 2. Related Work

The design of authentication schemes is one of the most important research aspects in IoT security. Sandeep et al. [16] proposed a dynamic identity-based authentication protocol for multi-server architecture that can resist several kinds of attacks by providing mutual authentication, anonymity, and session key agreement. However, it could not resist leak-of-verifier attack, impersonation attack, and stolen smart card attack. Then, Butun et al. [17] proposed a cloud-centric multi-level authentication-as-a-service approach. Since certification verification, which requires many asymmetric encryptions, was adopted in the authentication process [17], it is not suitable as a lightweight authentication scheme for smart homes. Ramos et al. [18] presented a set of elliptic curve cryptography optimizations for point and field arithmetic for the design and implementation of a security- and capability-based access control mechanism for smart objects. Shen et al. [12] mainly focused on an efficient multilayer authentication protocol and a secure session key generation method. Based on certificateless cryptography between two entities, they proposed a new certificateless authentication protocol with no pairings. In the protocol, the elliptic curve cryptography algorithm is used to provide low computational cost with high security. However, there are too many point multiplications in this scheme, which incurs high computational costs. Shen et al. [19] described a cloud-aided lightweight certificateless authentication protocol for wireless body area networks. The protocol achieves mutual authentication in the insecure channel by computing a message authentication code.

In smart homes, to accomplish a specific task, many devices would work cooperatively via wireless communication; thus, they must communicate with each other in a trusted and uncompromised module. Chifor et al. [20] proposed a lightweight authorization scheme for smart homes. In this scheme, a robust security authorization solution is implemented by the smart phone component with a password-less authentication protocol using the fast identity online model. Likewise, a lightweight and real-time protocol for anonymous authentication was proposed to protect data in wireless sensor network [21] that can guarantee anonymity, intractability, and forward and backward security.

Key agreement is another important problem that needs be addressed in IoT systems. Users and smart devices must establish a secure communication channel, in which a shared key is used to encrypt the transmission data. Rathore, M.M. [22] adopted signature to guarantee the data integrity in the session key exchange phase, which will lead to a heavy computation cost for IoT systems. Based on Datagram Transport Layer Security (DTLS) handshake, Moosavi et al. [23] proposed an end-to-end security scheme for mobility enabled healthcare Internet of Things, which is not an efficient solution for smart homes. In [24], the authors proved that the ECDH algorithm is more suitable for IoT environment than RSA algorithm through power and performance analysis. Song et al. [25] proposed an improved energy efficient, secure, and privacy-preserving communication protocol. In the protocol, the shared key is generated by a chaotic system. The chaotic system is characterized by its extreme sensitivity to the initial conditions and its topologically mixing property, but the performance of this scheme depends on the accuracy of time synchronization in smart homes. Shen et al. [13] focused on the security of uploading data in the smart home system. The authors introduced a secure key agreement scheme based on an improved ECDH algorithm, which ensures that the cloud validates the data integrity while preventing malicious home gateways form monitoring or modifying the data.

In the aforementioned papers, some methods provide a secure authentication or key agreement protocol by adopting asymmetric signatures, which incur high computation costs. Other methods are mainly based on ECDH schemes to realize the key exchange. Inspired by them, we constructed a lightweight authentication and key agreement protocol for smart homes based on one-way hash authentication and the CRT and ECDH combination scheme.

## 3. Preliminaries

In this section, a brief system model for smart home is provided first. The design goals are discussed and some reasonable system assumptions are made. Then, some related algorithms are introduced.

### 3.1. System Model

**Figure 1:**
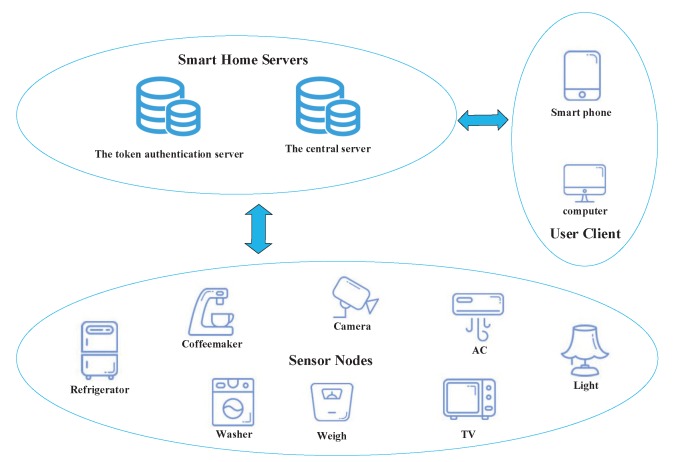
The proposed smart home architecture.

A smart home network can be regarded as a network of many sensor devices with constrained computation capacity and low memory storage. These devices are intelligent to provide convenient services to people, for instance, automatically adjusting temperature to make the home environment more livable, triggering a ceiling fan to switch on when someone walks into a room, and controlling lights to switch off when someone leaves. The smart home architecture model considered in this paper is shown in Figure 1, which involves three main entities: sensor nodes, smart home servers, and user clients. These entities are described as follows:

Sensor Nodes (SNs): Sensor nodes can collect monitor information from some related devices and send these data to the central server periodically.

Smart Home Servers (SHs): Smart home servers are composed of a central server (CS) and a token authentication server (TAS). The CS is mainly used to manage the sensor nodes and handle the user application requests. The TAS is mainly responsible for the token distribution and new device registration for the smart home system. The TAS is also used to store the fundamental registration information of sensor nodes, which is the precondition of the key agreement process.

User Clients: The user client provides an interface for users to communicate with a smart home system. It ensures the users can configure, monitor, and control smart home devices.

### 3.2. Design Goals

Our two main design goals in this study were:

(1) Lightweight secure authentication: The trade-off between security and performance should be considered when designing the authentication scheme for smart home devices.

(2) A secure key exchange: The integrity of the transmitted data and security of key exchange should be guaranteed. The security features can be guaranteed by the key agreement combination algorithm. In this paper, we adopt the CRT to strength the security of the key agreement. The shared key is used to encrypt transmission data. The protocol should be efficient.

### 3.3. System Assumptions

Some reasonable assumptions are outlined as follows.

Both the CS and TAS can be fully trusted. Note that both of them have large computation and storage capabilities. We assume that the communication channel between TAS and CS is secure; thus, the CS can query some necessary information from the TAS. We assume that the communication between the TAS and the sensor node is protected via an encrypted channel, which can be regarded as a secure channel. Here, the main problem we consider is the authentication and communication security between sensor nodes and the central server, in which the sensor nodes are not trusted entities and have limited computational and storage capabilities.

In smart homes, all sensor nodes are pre-loaded with a unique identity $ID_{SN}$. A secure symmetrical encryption key between the TAS and each sensor node is also fixed in both entities to secure the transmission of a small number of sensitive parameters. Note that physical unclonable function technology can be used to pre-load these identities and symmetrical encryption keys for heterogeneous devices.

To better illustrate our protocol, Table 1 shows all the symbols and notations used in the proposed protocol.

### 3.4. Related Algorithms

In this section, we briefly introduce the Chinese Remainder Theory (CRT) [26] and ECDH algorithm [27]. Both are fundamental to the key agreement combination method.

#### 3.4.1. Chinese Remainder Theory

In number theory, the CRT states that we can uniquely solve any pair of congruence equations, which enabled us to devise an efficient parameter agreement scheme in smart homes. The details are provided as follows

Suppose that $m_{1},m_{2}\ldots ,m_{k}$ are pairwise relatively prime positive integers, and $a_{1},a_{2}\ldots ,a_{k}$ are integers where $0<a_{i}<m_{i}$. Then, the system of any pair of congruences, $x\equiv a_{i}\;\mathrm{mod}\;m_{i}$ for 1≤i≤k, has a unique solution modulo $M=m_{1}\times m_{2}\times \ldots \times m_{k}$, which is given by:

$$x\equiv a_{1}y_{1}M_{1}+a_{2}y_{2}M_{2}+\ldots +a_{k}y_{k}M_{k}\left(\mathrm{mod}M\right)$$

where $M_{i}=M/m_{i}$ and $y_{i}\equiv {M_{i}}^{-1}\;\mathrm{mod}\;m_{i}$ for 1≤i≤k.

#### 3.4.2. ECDH

ECDH is a key agreement protocol that enables two entities, e.g. Alice and Bob, to share a secret key using elliptic curve Diffie–Hellman.

(1) Alice and Bob choose a common elliptic curve *E* over a prime field GF(P), where *P* is a base point.

(2) Alice chooses an integer *a*, which is a secret key and not shared with anyone. Then, Alice performs point multiplication and calculates the public key $PU_{a}=aP$, and sends $PU_{a}$ to Bob.

(3) Bob also selects an integer *b*, which is his private key, and then calculates $PU_{b}=bP$ by point multiplication and sends $PU_{b}$ to Alice. Alice computes aPb=abP. This is achieved by point multiplication of Alice’s secret key with Bob’s shared key. Bob performs point multiplication between bob’s private key and Alice’s public key and computes bPa=abP. Thus, both sides can obtain one secure shared key.

## 4. The Proposed Protocol

In this section, a lightweight one-way authentication and key agreement protocol based on CRT and ECDH is presented. Our scheme contains two phases: a registration phase and a key agreement phase.

### 4.1. Registration Phase

Since the TAS is a fully trusted entity in the system, it is reasonable to assume that the TAS bootstraps the whole system. In the registration phase, the sensor nodes can register to the TAS via their own initialization information. Firstly, the sensor node sends their identity to the TAS. After the TAS receives the identity of the sensor node, the TAS verifies whether this identity is legal by querying the sensor node information database maintained by the TAS. If the TAS finds that the sensor node identity is not registered in the database, the TAS will compute the unique token code $token_{SN}$ for the sensor node. The unique token code $token_{SN}$ can be computed by some related parameters, such as the sensor node identity ID, timestamp, and other server environment parameters. After that, the sensor node identity is labeled as registered. This ensures that the sensor node identity can only be used once and prevents illegal sensor nodes from registering to this smart home system. Before sending registration reply information to the sensor node, the TAS will choose two large security prime numbers, $m_{1}$ and $m_{2}$, and sends $m_{1}$, $m_{2}$, and $token_{SN}$ back to the sensor node. $m_{1}$ and $m_{2}$ are used to limit the value range of $a_{1},a_{2}$, and each sensor node can have a different pair of $\left(m_{1},m_{2}\right)$. The transmission of these sensitive paymasters, $m_{1}$, $m_{2}$, and $token_{SN}$, can occur in a predefined secure channel (see Section 3.3).

### 4.2. Key Agreement Phase

In the key agreement phase, the sensor node and the CS chooses an elliptic curve $E_{P}\left(a,b\right)$, $y^{2}=x^{3}+ax+b\;\mathrm{mod}\;P$, where *P* is a generator point on $E_{P}\left(a,b\right)$.

**Figure 2:**
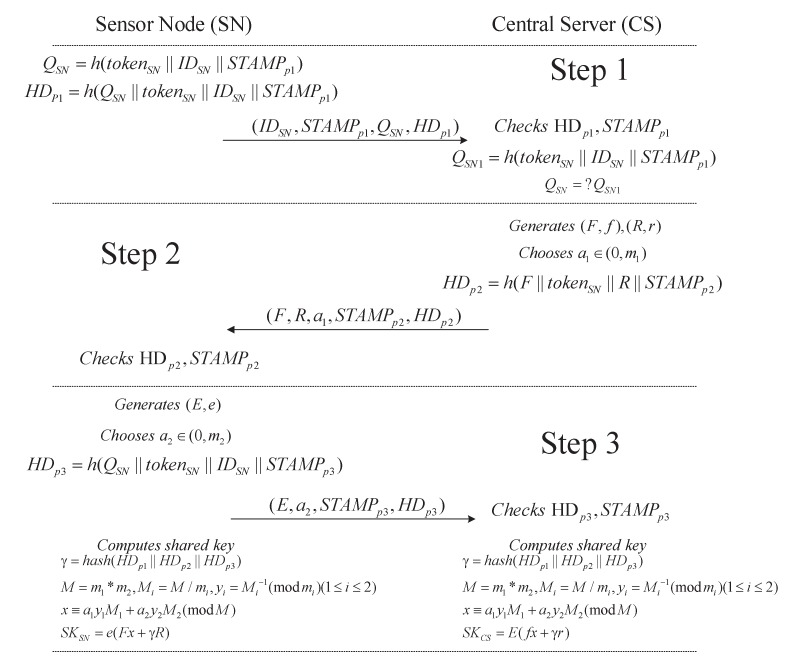
The key agreement protocol between the sensor node and the central server.

The key agreement phase generates a shared key between a sensor node (SN) and the CS. The entire key agreement process is illustrated in Figure 2.

Step 1: After the SN is registered, some initialization parameters are obtained, including the sensor node identity $ID_{SN}$, the authentication token $token_{SN}$, and the Chinese Remainder Theory parameters $m_{1}andm_{2}$. Then, the SN computes the most important authority message digest: $Q_{SN}=h(token_{SN}||ID_{SN}||STAMP_{p1})$ and the hash digest of the current package $HD_{p1}$, where the index variable p1 represents the first phase in key agreement. All these parameters, $Q_{SN},ID_{SN},STAMP_{p1},andHD_{p1}$, will be sent to the CS. After receiving these parameters, the CS computes $Q_{SN1}=h(token_{SN}||ID_{SN}||STAMP_{p1})$ and verifies whether the equation $\left(Q_{SN}=Q_{SN1}\right)$ makes sense. If this equation is not true, this key agreement step will be stopped immediately.

Step 2: After successful verification, the CS generates two pairs of public keys and private keys: (F,f) and (R,r). Subsequently, the CS chooses a random number $a_{1}\left(0<a_{1}<m_{1}\right)$. Then, the CS sends parameters *F*, *R*, $a_{1}$, $STAMPP_{p2}$, and $HD_{p2}$ to the sensor node.

Step 3: When the sensor node receives the parameters from the CS, the sensor node generates a pair of public and private keys (E,e), chooses a random number $a_{2}\left(0<a_{2}<m_{2}\right)$, and then computes $HD_{P3}=h(E||a_{2}||token_{SN}||STAMP_{p3})$. Afterwards, the sensor node sends parameters *E*, $a_{2}$, $STAMPP_{p3}$, and $HD_{p3}$ to the central server. Finally, both sides can use known parameters to compute the same shared key.

The correct proof of the proposed scheme is shown below.

The sensor node can compute the shared key as follows:

$$\gamma =hash(HD_{p1}||HD_{p2}||HD_{p3})$$

$$M=m_{1}*m_{2},M_{i}=M/m_{i},y_{i}={M_{i}}^{-1}\left(\;\mathrm{mod}\;m_{i}\right)\left(1\leq i\leq 2\right)$$

$$x\equiv a_{1}y_{1}M_{1}+a_{2}y_{2}M_{2}\left(\;\mathrm{mod}\;M\right)$$

$$SK_{SN}=e\left(Fx+\gamma R\right)$$

The central server can compute the shared key as follows:

$$\gamma =hash(HD_{p1}||HD_{p2}||HD_{p3})$$

$$M=m_{1}*m_{2},M_{i}=M/m_{i},y_{i}={M_{i}}^{-1}\left(\;\mathrm{mod}\;m_{i}\right)\left(1\leq i\leq 2\right)$$

$$x\equiv a_{1}y_{1}M_{1}+a_{2}y_{2}M_{2}\left(\;\mathrm{mod}\;M\right)$$

$$SK_{CS}=E\left(fx+\gamma r\right)$$

Hence, the sensor node and the central server can obtain the same shared key in our proposed protocol. A strong correlation exists between the shared key and the parameters in the key agreement. As shown in Figure 2, by incorporating the CRT into ECDH, our proposed protocol increases the complexity and security of the key agreement process.

## 5. Security Analysis and Performance Evaluation

In this section, the security analysis and performance evaluation of proposed protocol are presented, and the conducted experiments are outlined to show the efficiency of the protocol.

### 5.1. Security Analysis

(1) Authentication. In the proposed protocol, one-way authentication is used for the central server to verify the identities of the sensor nodes. The sensor node can compute $Q_{SN}=h(token_{SN}||ID_{SN}||STAMP_{p1})$, and send the essential parameters $Q_{SN}$, $ID_{SN}$, and $STAMP_{p1}$ to the central server. If $Q_{SN}=Q_{SN1}$, the sensor node is identified by the central server. The central sever can obtain the token of the sensor node from TAS via a secure channel. The adversary could not obtain the security parameter: the sensor node token. Therefore, the authentication method is secure.

(2) Data Integrity. The data integrity is guaranteed by tagging the hash digest to the packet. The adversary can neither obtain the legal token nor modify the hash digest. Hence, this method can guarantee the data integrity.

(3) Resistance to replay attack. A replay attack (also known as playback attack) is a form of network attack in which a valid data packet is maliciously or fraudulently repeated or delayed. At time t + 1, the attacker might replay a data packet that was captured at time t. When the attacker sends the captured packet to the central server, the central server mistakenly thinks the packet was sent by a legal sensor node at time t + 1; in this way, the attacker is disguised as a sensor node. In the proposed solution, this attack is resisted by introducing timestamp and token in each communication packet. These token and timestamp are hashed and sent to the receiver, thus the packets cannot be altered. Thus, if the packet is replayed again, the receiver will detect the modification by verifying the hash digest. Hence, no replay attack can be performed.

(4) Resistance to man-in-the-middle attack. Man-in-the-middle attack is generally performed to obtain access to the information sent from source to the destination. The adversary quietly relays and possibly alters the communication information between two entities who believe that they are directly communicating with each other. Our proposed scheme resists this attack by providing the hash code check and timestamp verification. The adversary cannot generate one legal hash code message. The adversary would not obtain the right token, which is the most important part of hash code generation parameter. Hence, the method ensures that data cannot be tampered with by any adversary in the protocol. In the man-in-the-middle attack, an adversary may only tamper with all the information in the packet, adding their information message to the packet and then sending it to the central server presenting as a legal sensor node. However, the adversary cannot pretend to be a legal sensor node if they cannot obtain the correct sensor node token. In other words, the proposed protocol can resist man-in-the-middle attacks.

(5) The security of the key agreement combination method. This combination method is based on CRT and ECDH. CRT can negotiate the same parameters with two different congruence formulas. Then, the same parameters are used to compute the final shared key with the ECDH algorithm. The combination of the key agreement scheme is difficult to crack, thus this construction method can dramatically strengthen the protocol security. Note that, in our protocol, $token_{SN}$ is an important security parameter, which might suffer from the brute-force attack. To resist such attacks, the only thing we can do is increase the size of $token_{SN}$, but resisting quantum computing attacks, as in any other crypto-system, remains challenging.

### 5.2. Performance Evaluation

This protocol is based on the elliptic curve cryptography algorithm. Elliptic curve cryptography is superior in terms of short key size, low computation, and high security. In this proposed protocol, the key agreement phase contains three data exchanges. The first data exchange is used for authentication, and the last two data exchanges are used for the key agreement. In this subsection, the performance evaluation is presented from three aspects: computational complexity, memory size, and communication overhead.

(1) Computational Complexity. For the key agreement process, the sensor node needs to conduct an ordering data operation that contains authentication, parameter exchange, and shared key computation. The computational complexity comparison is presented in Table 2. The main computational overhead is composed of four multiplications and four hash functions. Compared with other lightweight schemes, the computational cost of our scheme marginally increases. Our scheme sacrifices low computational resource for increased security.

(2) Memory Size:.The memory cost of the proposed scheme was evaluated by computing the length of the message sent by the sensor node and the central server. The memory cost is discussed below. The proposed protocol consists of three steps.

In the first step, the sensor node generates $Q_{SN}=h(token_{SN}||ID_{SN}||STAMP_{p1})$, which is 160 bits long. In the second step, the central server verifies the identity of the sensor node by computing the authentication message $Q_{SN1}=h(token_{SN}||ID_{SN}||STAMP_{p1})$. Then, the central server generates the CRT parameter $a_{1}$ and two 256-bit key pairs. In the final step, the sensor node receives the parameters from the central server; the sensor node also chooses a random number $a_{2}$ and generates a key pair. The sensor node sends $a_{1}$ and the public key to the central server.

As mentioned above, the sensor nodes are equipped with limited computing power, storage, and communication modules. Therefore, we adopt lightweight combination method based on Chinese Remainder Theorem and ECDH to generate the shared key. Assume that the eclipse curve key length is 256 bits. Table 3 shows the length of every parameter used in our scheme. The detail analysis of memory cost is demonstrated from two sides: the sensor node side and the central server side.

On the sensor node side, the memory cost of the sensor node includes $Q_{SN}=h(token_{SN}||ID_{SN}||STAMP_{p1})$, one public/private key pair, four random numbers (10 bits), $ID_{SN},token_{SN}$, two public CS keys, three hash digests, and three timestamps. Hence, the total memory cost is 2265 bits.

On the central server side, the central server receives $Q_{SN}$, $ID_{SN}$, $STAMP_{p1}$, and $HD_{p1}=h(Q_{SN}||token_{SN}||ID_{SN}||STAMP_{p1})$ and computes $Q_{SN1}=h(token_{SN}||ID_{SN}||STAMP_{p1})$ and $HD_{p1}$, which costs 791 bits in total. Then, the central server needs to store some variables, including two public/private key pairs, the public key of the sensor node, four numbers, two hash digests, and two timestamps, totalling 1978 bits.

Hence, the total memory cost of the server and the sensor node is 2769 bits.

(3) Communication Overhead. In this protocol, the communication overhead in the key agreement period is considered. The size of three packets is illustrated in Table 4. The first packet consists of an authentication message $Q_{SN}$, $ID_{SN}$, and the message digest $HD_{p1}$. Therefore, the first packet size is 525 bits. The second packet includes $a_{1}$, *F*, *R*, $STAMP_{p2}$, and one hash digest, thus it is 601 bits. The last packet contains $a_{2}$, *E*, $STAMP_{p3}$ and one hash digest, so is 439 bits. Hence, the total communication overhead of these three packets is 1292 bits, which means that our proposed protocol is lightweight and efficient.

In this paper, one-way authentication is adopted to reduce resource consumption, which is suitable for the smart home environment. Compared with the mutual authentication, one-way authentication can reduce the authentication overhead by half.

In summary, we aimed to resolve several security issues existing in the authentication and key agreement for smart homes, while simultaneously meeting the lightweight and efficient requirements of the protocol. A comprehensive comparison between our protocol and some others schemes is provided in Table 5. The protocols presented by Liu, Y. [15], Butun, I. [17] and Rathore, M.M. [22] use the asymmetric signature algorithm to ensure entity mutual authentication; however, they require considerably more computational and storage resources than ours. Previous authors [29] proposed an ECDH-based security model for ESP8266, but the implementation of the ECDH algorithm on ESP8266 devices does not provide a customized design for smart homes. Other authors [24] proposed an ECDH agreement algorithm for image encryption, which is not suitable for smart homes. The protocol proposed in [13] is lightweight and smart-home-supported; however, it can neither guarantee the data integrity nor provide the authentication. Park, K. [14] described a lightweight protocol with authentication function, but it can resist neither replay attacks nor man-in-the-middle attacks. Therefore, considering the trade-off between security and performance, our protocol is more suitable for smart home scenarios.

### 5.3. Experiments

In this subsection, the experiments used to verify the efficiency of our protocol are outlined. In the experiments, the sensor nodes environment was implemented based on Arduino, which is a convenient and flexible open source electronic prototype platform. Here, the WiFi connection chip ESP8266 was adopted as the sensor node. ESP8266 is a kind of cost-effective and low-power WiFi chip that can be used to easily set up a WiFi network for a smart home. It can be used in many smart home devices, such as wireless lamp, television, and curtain switches. We used an open source library, Arduino Cryptography Library (https://github.com/rweather/arduinolibs) as the crypto library, which is popular for IoT development. The server environment was simulated on a JAVA web platform. Our protocol model consisted of an authentication server, a central server, and some sensor nodes. There were two main communication channels: one between sensor nodes and the TAS and the other between sensor nodes and CS.

**Figure 3:**
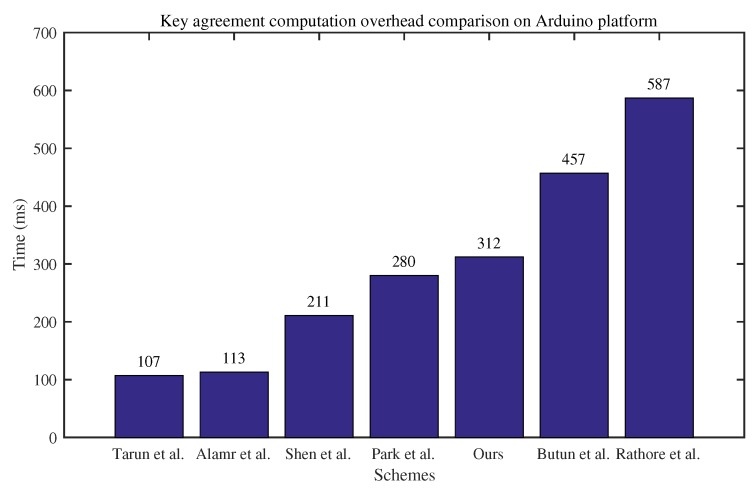
Computation overhead comparison between different protocols on Arduino platform.

In the first group of experiments, the key agreement computation overhead of different protocols was applied on the Arduino platform. Note that each result is the average of 100 tests. As shown in Figure 3, the time cost of our protocol is larger than the protocols proposed by Tarun et al. [24], Alamr et al. [28], Shen et al. [13], and Park et al. [14], but significantly less than the protocols proposed by Butun et al. [17] and Rathore et al. [22]. Considering the security of our protocol, the computation cost of key agreement is acceptable for many smart home systems.

**Figure 4:**
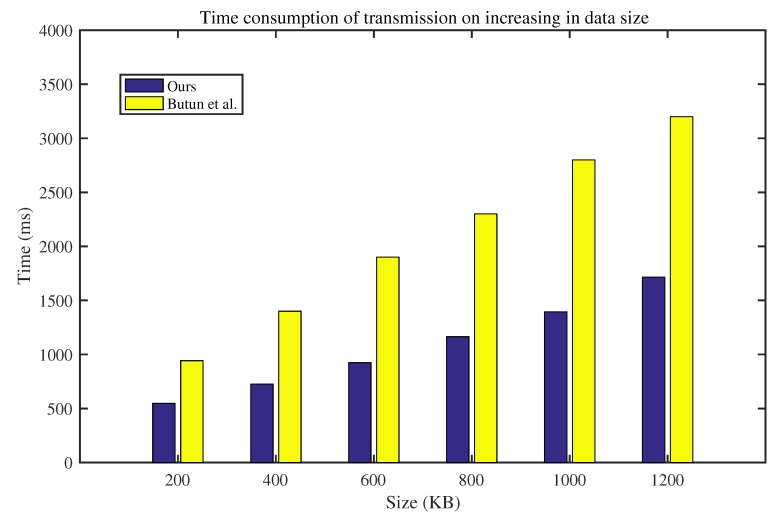
Time consumption of transmission with increasing data size.

In the second group of experiments, we examined how the time cost of data transmission changes with the increase in data size. The time cost includes the authentication time, the key agreement time, encryption time, and decryption time. Here, advanced encryption standard was adopted as the encryption algorithm. The average time cost for 100 tests is shown in Figure 4. With the increase in data size, the time consumption of our protocol is much lower than Butun’s protocol [14]. This experiment showed that our lightweight protocol can work in the smart home environment better than Butun’s protocol.

## 6. Conclusions

In this paper, a lightweight one-way authentication and key agreement protocol based on CRT and ECDH in smart homes is presented. Firstly, one-way hash authentication, which provides enough security for the authentication, is adopted to reduce computational and memory storage costs. Then, the Chinese Remainder Theorem is introduced to enhance the security of the original ECDH key agreement. Finally, the security analysis, performance analysis, and experiments showed that our protocol is secure and more suitable for the constrained devices in smart homes than some existing protocols. As a result, our proposed protocol provides high security and low communication and computation costs, thus can be implemented in smart home systems.

## Figures and Tables

**Table 1:** Symbols and notations used in the protocol.

Notations	Description
SN	Sensor node
CS	Central server
TAS	Token authentication server
$ID_{X}$	Identity of *X*
$token_{X}$	Token of *X*
h(X)	Hash function applied on *X*
*P*	Base point of the elliptic curve
$m_{i}$	A large number that limits the value range of $a_{i}$
$a_{i}$	A random number of entity *i*, $0<a_{i}<m_{i}$
$STAMP_{pi}$	Time stamp on the phase pi, where i=1,2
$SK_{SN,CS}$	Shared key between SN and CS
$HD_{pi}$	Hash digest of the phase pi, where i=1,2,

**Table 2:** Comparison of computational costs, where H represents hash function and M represents point multiplication over the elliptic curve.

Algorithm	Computation Cost of Each Node
Shen et al. [13]	3M + 2H
Park et al. [14]	3M + 5H
Rathore et al. [22]	4H + 9M
Tarun et al. [24]	2M
Alamr et al. [28]	2M
Ours	4M + 4H

**Table 3:** The length of parameters used in our proposed scheme.

Parameter	Length (bits)
ECC key	256
Hash digest	160
CRT parameter	10
Device ID	10
Device token	256
Timestamp	13

**Table 4:** Packet size (bits).

First Packet Size	Second Packet Size	Last Packet Size
525	601	439

**Table 5:** A comprehensive comparison between different protocols.

Protocol	Lightweight	Authentication	Data Integrity	Smart Home Support	Replay Attack Resistance	Man-in-the-Middle Attack Resistance
Shen et al. [13]	Yes	No	No	Yes	Yes	Yes
Park et al. [14]	Yes	Yes	No	No	No	No
Liu et al. [15]	No	Yes	Yes	No	No	No
Butun et al. [17]	No	Yes	Yes	No	No	No
Rathore et al. [22]	No	Yes	Yes	No	Yes	Yes
Tarun et al. [24]	Yes	No	Yes	No	No	No
Ravi et al. [29]	Yes	No	No	No	No	No
Ours	Yes	Yes	Yes	Yes	Yes	Yes
